# Supplementary material for: ZnO-AuxCu1−x Alloy and ZnO-AuxAl1−x Alloy Vertically Aligned Nanocomposites for Low-Loss Plasmonic Metamaterials
Source: Molecules. 2022 Mar 9;27(6):1785. doi: 10.3390/molecules27061785 (PMC8948785; doi:10.3390/molecules27061785)
Supplement: Supplementary file 1 [file molecules-27-01785-s001.zip › molecules-1520540-supplementary.pdf]

Supporting information:

ZnO-Au_xCu_{1-x} alloy and ZnO-Au_xAl_{1-x} alloy vertically-aligned nanocomposites for low-loss plasmonic metamaterials

Robynne L. Paldi[a], Juanjuan Lu[a], Yash Pachaury[a], Zihao He[b], Xinghang Zhang[a], Anter El-Azab[a], Aleem Siddiqui[c], Haiyan Wang[a,b,*]

[a] School of Materials Engineering, Purdue University, West Lafayette, Indiana 47907, United States

[b] School of Electrical and Computer Engineering, Purdue University, West Lafayette, Indiana 47907, United States

[c] Sandia National Laboratory, Albuquerque, New Mexico, USA

*corresponding author: Haiyan Wang, hwang00@purdue.edu

Keywords: ZnO, Au, vertically aligned nanocomposite, low-loss, metamaterials, Cu, Al

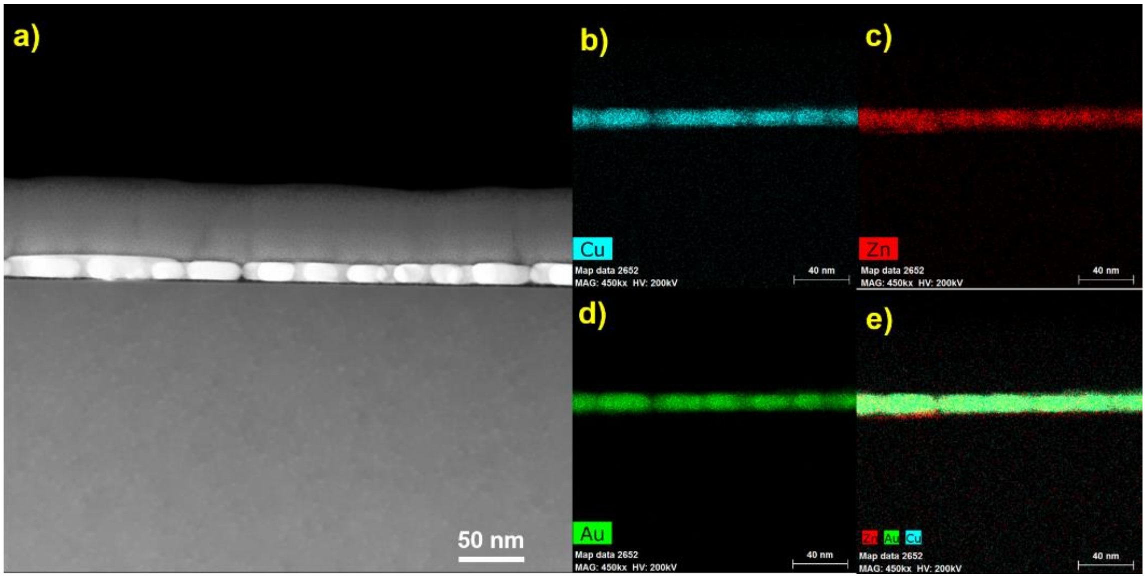

Figure S1 ZnO-Au_xCu_{1-x} one-step growth. a) STEM cross-section. Elemental EDS-mapping of b) Cu, c) Zn, d) Au, and e) combined.

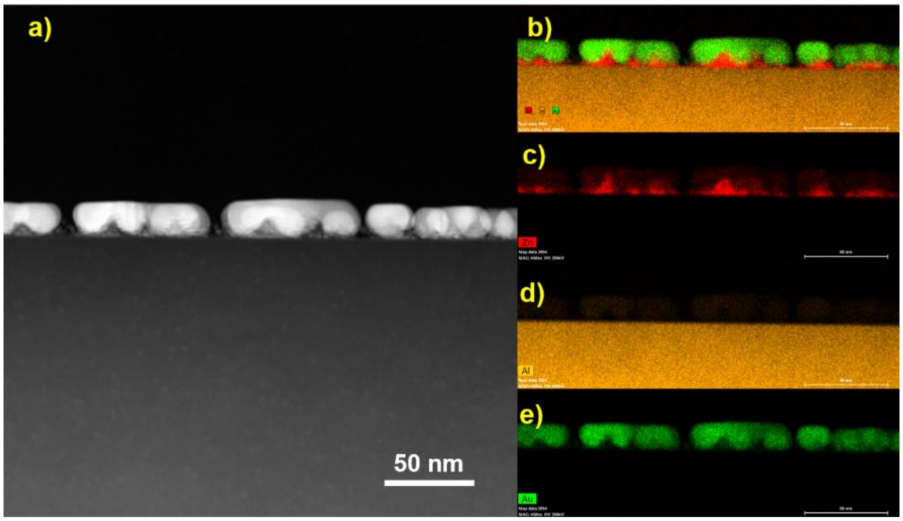

Figure S2 Deposition of ZnO-Au_xAl_{1-x} through one-step growth. a) STEM cross-section. Elemental EDS-mapping of b) combined, c) Zn, d) Al, and e) Au.

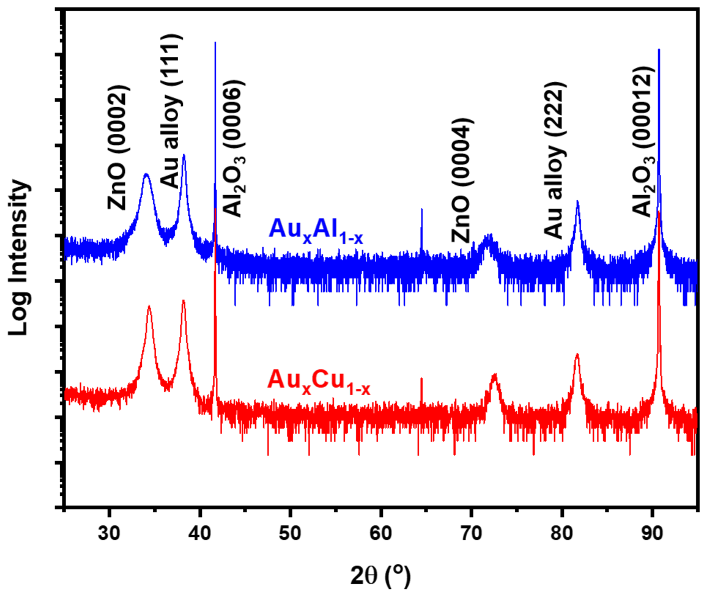

Figure S3 X-ray diffraction scans of θ-2θ performed for each alloy film.

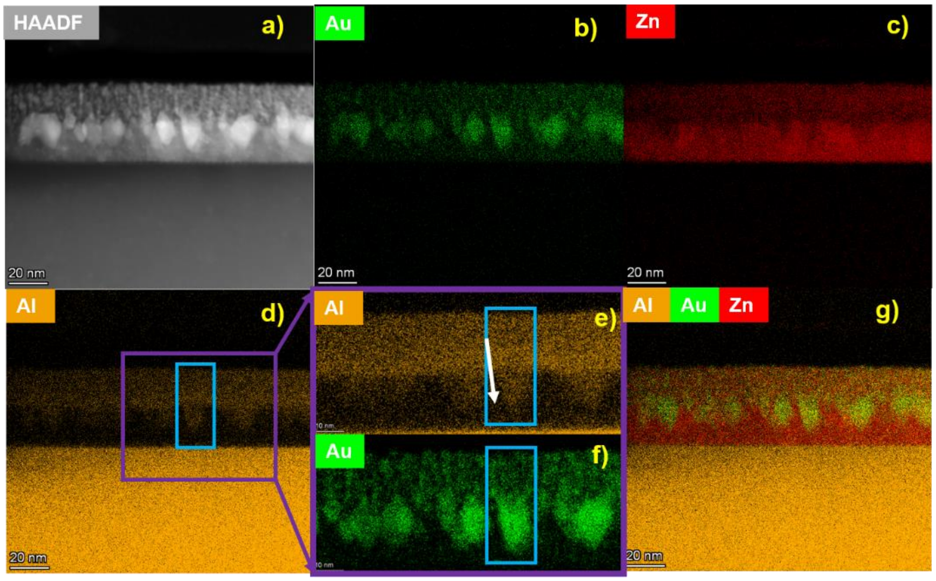

Figure S4 ZnO-Al_xAu_{1-x} cross section. a.) HAADF STEM cross-section micrograph. EDS-mapping of b.) Au, c.) Zn, d.) Al. e.) Zoomed in Al EDS map showing diffusion. f.) Zoomed in Au EDS-map. g.) Combined EDS mapping.

Unlike the ZnO-Au_xCu_{1-x} VAN, the two-step growth did not form a cohesive thin film growth. When Al is introduced to the growth, it causes particle formation in the second layer. EDS-mapping was performed on the ZnO-Au_xAl_{1-x} with mapping for Au, Zn, and Al shown in Figure S4 b, c, d respectively. Interestingly, in the alloy layer the Al diffuses throughout the layer, possibly forming both an Au_xAl_{1-x} nanoalloy and Al-doped ZnO. The Al also appears to diffuse down into the Au pillars in the seed-layer. A zoomed in version of one of the pillars is shown with Al and Au mapping in Figure S4e and f, respectively. The mapping of Al overlaps with that of the Au pillar, possibly indicating the diffusion of Al into Au and subsequent formation of Au_xAl_{1-x} nanoalloy. A combined mapping is depicted in Figure S4g of Al, Au, and Zn. Moreover, characterization of the plan-view was also performed including STEM and EDS-mapping and depicted in Figure

S5. Results confirm the diffusion of Al into the ZnO matrix and possible formation of Al-doped ZnO.

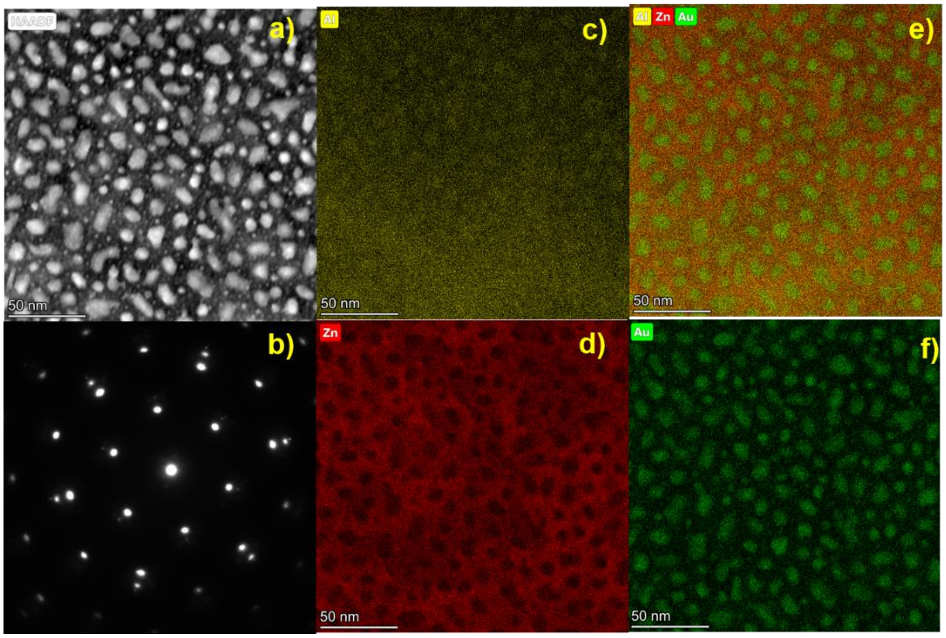

Figure S5 Plan-view of ZnO-Au_xAl_{1-x}. a) Typical HAADF image, b) plan-view SAED image. Elemental EDS-mapping of c) Al, d) Zn, e) Au, and f) combined.

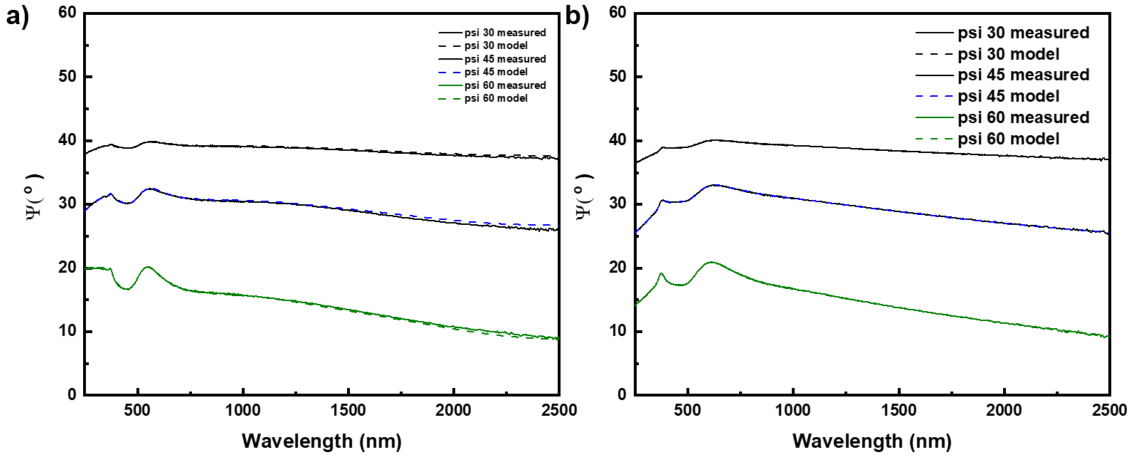

Figure S6 Measured and model fit of experimental psi for a) ZnO-Au_xCu_{1-x} and b) ZnO-Au_xAl_{1-x}.

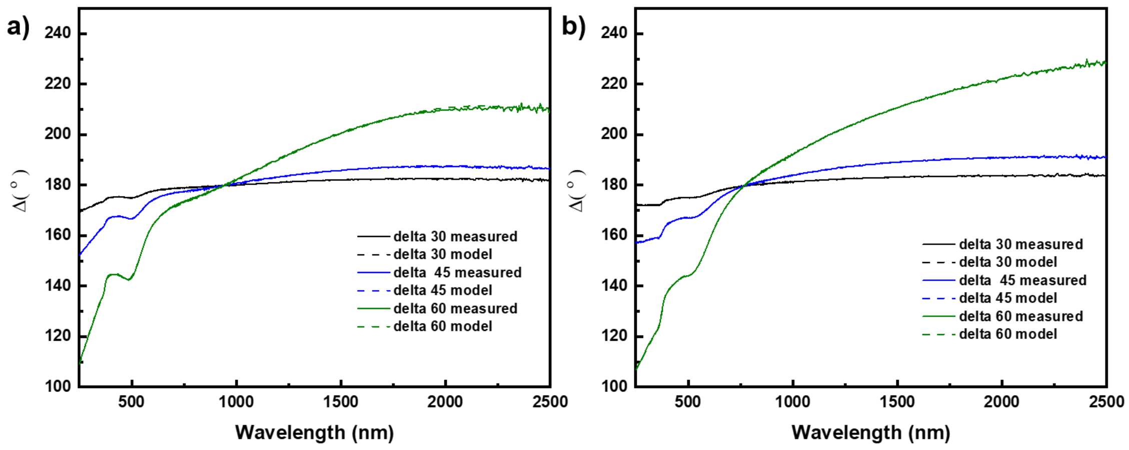

Figure S7 Measured delta from ellipsometry for a) ZnO-Au_xCu_{1-x} and b) ZnO-Au_xAl_{1-x}
